# Yield of close contact tracing using two different programmatic approaches from tuberculosis index cases: a retrospective quasi-experimental study

**DOI:** 10.1186/1471-2466-14-133

**Published:** 2014-08-07

**Authors:** Carla Loredo, Michelle Cailleaux–Cezar, Anne Efron, Fernanda Carvalho Queiroz de Mello, Marcus Barreto Conde

**Affiliations:** 1 Institute of Thoracic Diseases of Federal University of Rio de Janeiro, Rio de Janeiro, Brazil; 2 Center for Tuberculosis Research, Johns Hopkins University, Baltimore, Maryland, USA; 3 Hospital Universitário Clementino Fraga Filho, Cidade Universitária, Rua Professor Rodolpho Paulo Rocco n° 255, 6° andar, Rio de Janeiro 21941-913, Brazil

**Keywords:** Tuberculosis, Contact, Diagnosis, Latent tuberculosis

## Abstract

**Background:**

Being a contact of a pulmonary tuberculosis (TB) case is a risk factor for active and latent TB. The objective of this study is to determine the contact tracing yield using two different programmatic definitions of close contact in the city of Rio de Janeiro, Brazil.

**Methods:**

This is a retrospective quasi-experimental study. Data were obtained by reviewing the medical records from TB index cases and their close contacts admitted to the Outpatient TB Clinic of the Institute of Thoracic Diseases, University of Rio de Janeiro. From January 2001 to December 2004, a close contact was defined as an individual who shared an enclosed space with a TB index case for a total period of ≥ 100 hours, whereas from January 2005 to December 2008 the definition of close contact was changed to an individual who shared an enclosed space with a TB index case ≥ 4 hours a week. The primary outcome of this study was newly diagnosed pulmonary TB cases and the secondary outcome was the prevalence of latent TB infection (LTBI) among close contacts during both periods.

**Results:**

From 2001–2004, 810 close contacts from 257 index cases were evaluated and the prevalence of active TB and LTBI were 2% (16/810) and 62% (496/794), respectively. From 2005–2008, 1,310 close contacts from 369 index cases were identified and the prevalence of active TB and LTBI were 2.7% (35/1,310) and 69% (877/1,275), respectively. There was not a statically significant difference in the detection of active TB (p = 0.3) between the 2 time periods, but the detection of LTBI was significant higher (p = 0.003). The number needed to screen (contacts/new cases) decreased from 50 to 37 and the number need to contact trace (index cases/new cases) decreased from 16 to 10 from 2001–2004 to 2005–2008.

**Conclusion:**

In conclusion, the findings of this study suggest that the less conservative definition of TB close contacts (sharing space ≥ 4 h/week) can be a helpful tool for increasing the rate of diagnosis for newly active pulmonary TB cases and for the detection of LTBI among contacts of active pulmonary TB cases.

## Background

It is internationally recognized that children under five years of age and persons living with human immunodeficiency virus (PLHIV) who were exposed to infectious cases of tuberculosis (TB) must be evaluated for active TB and considered for treatment of latent TB infection (LTBI) once active TB is excluded [1,2]. In Brazil, since 2009, the recommendation has been that close contacts of an infectious TB case, despite their age and HIV status, must be investigated for active TB and LTBI [3,4]. However, the different definitions utilized for the term “close contact of TB” can make the yield of contact tracing irregular among different settings [3,5-7]. In the Outpatient TB Clinic of the Institute of Thoracic Diseases (IDT) of the Federal University of Rio de Janeiro (UFRJ), Brazil, all contacts of TB index cases who shared and enclosed space for ≥ 100 hours in total used to be defined as a close contact and eligible to be investigated for active TB and for LTBI. However, in 2005, the TB Clinic of IDT/UFRJ reduced the exposure time for TB contacts from 100 total hours to ≥ 4 hours a week. This new definition for “close contacts” was based on the definition utilized by the Tuberculosis Trial Consortium (TBTC) from Center for Diseases Control and Prevention for the Study 26 Protocol (http://www.clinicaltrials.gov; NCT00164450), a clinical trial of 9 months of daily Isoniazid (INH) *versus* 12 weeks of weekly Rifapentine/INH for the prevention of TB. The Federal University of Rio de Janeiro (UFRJ) in collaboration with Johns Hopkins University was one of TBTC sites for Study 26 [8].

The purpose of this study was to compare the yield of contact tracing between the close contact definition utilized during the period of 2001–2004 (sharing space with an index case ≥ 100 hours total) with the definition utilized in the period 2005–2008 (sharing space with an index case ≥ 4 hours per week). Our hypothesis was that the change in the operational definition of a close contact to 4 hours/week would increase the detection of news cases of active TB and LTBI and reduce the number needed to contact trace (NNS).

## Methods

### Design

This is a quasi-experimental study with internal comparison.

### Setting

This study was conducted in the Outpatient TB Clinic of the IDT/UFRJ, a referral center for TB treatment and for TB Clinical Trials (former site 29, Hopkins-Brazil, from the Tuberculosis Trial Consortium of Center for Diseases Control and Prevention) in Rio de Janeiro, Brazil. The incidence rate of TB in Rio de Janeiro city during the first period of the study ranged from 94/100,000 (2001) to 85/100,000 habitants (2004) and during the second period it ranged from 80/100,000 (2005) to 70/100,000 habitants (2008) [9].

### Subject selection and data collection

The chart number of all TB cases and their contacts that were admitted to the TB Clinic of IDT/UFRJ between April 1^st^, 2001 to December 30^th^, 2008 were obtained from the register book. A data collection instrument was pre-tested and modified during a pilot study conducted in December 2008 with 20 charts (data not shown). The following data were recorded from the chart review: results from acid fast bacilli (AFB); TB culture; tuberculin skin test (TST); chest X ray (CXR); demographic data (sex, age); smoking status; alcohol consumption; use of illicit drugs; presence of diabetes; prior TB treatment; institutionalization (prison, shelter, or nursing home) within last 3 years; and HIV status (patients were tested for HIV only if active TB had been diagnosed; contacts were not tested for HIV). All contacts of a TB index case that were evaluated for LTBI and/or active TB in the Outpatient TB Clinic of IDT/UFRJ were enrolled in this study. Exclusion criteria were subjects with unavailable records, contacts with prior active TB treatment, missing results from the HIV test (index cases only), AFB, TST or culture and those with contaminated culture for *Mycobacterium tuberculosis* recorded in the chart.

As per the TB Clinic routine, during the interview in which the TB treatment is prescribed to an index case, a health care worker (HCW) explained to the patient the definition of a close contact (which was different on depending of the period evaluated, 2001–2004 or 2005–2008). Following this explanation, the HCW provided a printed invitation for close contacts ≥ 15 years old who were identified during the interview to attend a lecture conducted weekly in the TB Clinic. Because the Outpatient TB Clinic is an adult Clinic, the HCW instructed the index case to take all close contacts under 15 years of age to the Pediatric Outpatient Clinic of the University. The weekly lecture about TB was presented by a nurse who explained to all close contacts concepts about latent TB infection, active TB, diagnosis and treatment. After the lecture all contacts were invited to have a TST. Contacts with a positive TST or documented conversion as well as those with respiratory symptoms underwent a physical examination and had a CXR performed. Symptomatic subjects and/or those with altered CXR were instructed to provide two unsupervised sputum specimens for smear and *Mycobacterium tuberculosis* culture testing. Subjects with abnormal CXRs who were unable to provide sputum spontaneously underwent sputum induction with hypertonic saline solution. The TST was administered using the Mantoux technique and was read by a trained and certified (degree of intra-reader reliability > 95% and inter-reader reliability ≥ 80%) HCW. All testing was performed using purified protein derivative (PPD), RT23 (0.1 ml = 2 TU) (Staten Serum Institute, Copenhagen, Denmark) [10].

### Ethical approval

The study was approved by the Federal University of Rio de Janeiro Ethics Committee in July 2008.

### Definitions

Participants who reported smoking at least 100 cigarettes in their lifetime and who, at the time of survey, were smoking at least one day a week were defined as a current smoker; respondents who reported smoking less than 100 cigarettes in their lifetime and who, at the time of the survey, did not smoke at all were defined as former smoker; respondents who reported never having smoked any cigarette were defined as never smoking. Alcohol abuse was defined as daily consumption of at least 30 grams of alcohol for men and 24 grams for women. The definition of a close TB contact from 2001–2004 were all contacts of TB index cases who shared an enclosed space for ≥ 100 hours total. The definition of a close TB contact from 2005–2008 was those who shared an enclosed space ≥ 4 hours per week with a TB index case. A positive TST and conversion to a positive test were defined according to criteria of the American Thoracic Society and the Centers for Disease Control and Prevention [10,11]. LTBI cases were defined as a close contact with a positive TST or with a documented conversion. Confirmed active TB was defined as growth of *M. tuberculosis* in culture from respiratory specimen.

### Statistical methods

The primary outcome was newly diagnosed pulmonary TB during both periods (2001–2004 and 2005–2008). We also calculated the number of contacts needed to screen (NNS) to find one new case of active TB and the number need to contact trace (NNCT) [12,13]. The NNS is calculated by dividing the number of contacts screened by the number of new cases diagnosed [12]. The NNCT is calculated as the number of index cases divided by the number of new active TB cases [13]. The secondary outcome was the prevalence of LTBI among contacts during both periods (2001–2004 and 2005–2008).

### Statistical analysis

The Chi square test was used in the analysis of dichotomous variables and Fisher’s exact test was used when appropriate. The odds ratio (OR) of the association between independent variables and outcomes with 95% confidence interval (CI) was calculated. A p-value <0.05 was considered statistically significant. The results were analyzed using IBM SPSS Statistics 20.

## Results

**Figure 1:**
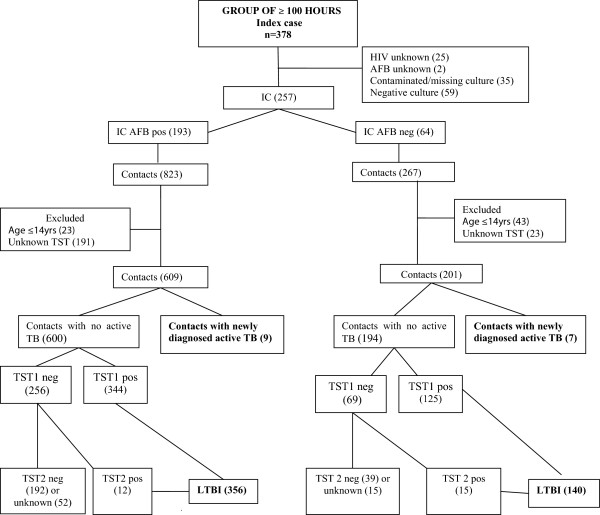
Contact evaluation of period 2001–2004.

**Figure 2:**
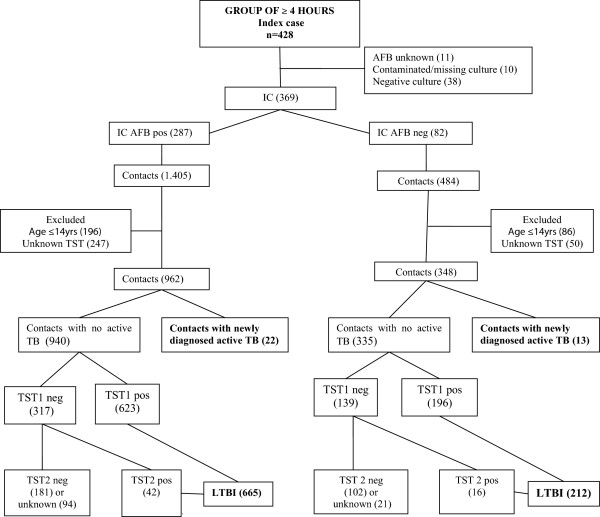
Contact evaluation of period 2005–2008.

Over a 92-month period, 806 patients with active pulmonary TB were admitted to the Outpatient TB Clinic of IDT/UFRJ. All medical records were reviewed and 180 active TB cases were excluded because the AFB results were unknown (n = 13), the M.tb culture results were missing or contaminated (n = 45), HIV status was unknown (n = 25) or the culture for *Mycobacterium tuberculosis* was negative (n = 97). From 626 active TB index cases included in the analysis (257 from 2001–2004 and 369 from 2005–2008), 2,979 contacts (1,090 from 2001–2004 and 1,889 from 2005–2008) were identified. Flow-charts showing the evaluation of contacts stratified by the AFB of the index case in both periods of study are presented in Figures 1 and 2. In the period from 2001–2004 (contacts shared ≥ 100 hours total) 810 contacts were evaluated. Two per cent (16/810) of those contacts were diagnosed as a newly active TB cases (detection rate of 1,975/100000) and 62% (496/794) as having LTBI. In the period from 2005–2008 (contacts shared ≥ 4 hours/week) 1,310 contacts were evaluated and 2.7% (35/1.310) had a diagnosis of active TB (detection rate of 2,442/100,000 habitants) and 69% (877/1,275) were diagnosed with LTBI. There was not a statically significant difference in the detection of active TB (16/810 or 2% *versus* 35/1310 or 2.7%, *p* = 0.3) between the 2 time periods, but the detection of LTBI was significant higher (496/794 or 62% *versus* 877/1275 or 69%, *p* = 0.003). However, the NNS for a newly diagnosed TB case decreased from 50 in the period from 2001–2004 to 37 in the period from 2005–2008. The same occurred with the NNCT, decreasing from 16 in the first period to 10 in the second. The characteristics of the study population are shown in Table 1. There were statistically significant differences between both samples in some variables, with slightly more males, household contacts, smokers and cases of diabetes in the first period (2001–2004). The association of different variables to the diagnosis of active TB and LTBI among contacts in both periods studied is presented in Tables 2 and 3, respectively. There was a statistically significant association between smoking and newly diagnosed active TB in the sample during the second period as well as those living in poor areas (slums) (Table 2). There was an association between a positive AFB in the index case and the diagnosis of LTBI in samples from both time periods. Smoking and alcohol abuse were associated with the diagnosis of LTBI only in the second period.

**Table 1:** Demographic characteristics of the study population stratified by period

		**Period**		
**Characteristics**		**2001-2004 n = (810)**	**2005-2008 n = (1310)**	***p value***
Age (mean ± DP)		38 (±16)	37 (±15)	0.2
Gender	Male	513 (63.6%)	762 (58.4%)	0.02
	Female	297 (36.4%)	548 (41.6%)	
Index case AFB positive	yes	609 (75. 2%)	962 (73. 4%)	0.3
	no	201 (24. 8%)	348 (26. 6%)	
Index case HIV positive	yes	4(0. 4%)	5 (0.3%)	0.9
	no	801 (99. 6%)	1284 (99. 7%)	
Contact households	yes	634 (78. 3%)	785 (59. 9%)	<0.001
	no	176 (21. 7%)	525 (40. 1%)	
Smoking	yes	82 (10.2%)	94 (7,2%)	0.01
	no	722 (89.8%)	1210 (92,8%)	
Use of injectable drugs	yes	2 (0. 2%)	12 (0.9%)	0.1
	no	801 (99.8%)	1286 (99. 1%)	
Use of non-injectable drugs	yes	17 (2.1%)	23 (1.8%)	0.6
	no	786 (97.9%)	1281 (98.2%)	
Residing in poor areas(slum)	yes	121 (16.0%)	216 (18.2%)	0.2
	no	635 (84.0%)	971 (81.8%)	
Prisoners/previous hospitalization	yes	28 (3.5%)	36 (2.8%)	0.4
	no	776 (96.5%)	1267 (97.2%)	
Diabetes *mellitus*	yes	22 (2.7%)	16 (1.2%)	0. 01
	no	783 (97.3%)	1294 (98.8%)	
Alcohol abuse	yes	19 (2. 4%)	45 (3.4%)	0.2
	no	786 (97. 6%)	1262 (96.5%)	
Use of immunosuppressive drugs	yes	4 (0. 5%)	4 (0.3%)	0.4
	no	801 (99.5%)	1299 (99.7%)	

Statistical test used for all variables was Chi-square, except for comparing age mean (Student).

**Table 2:** Variables associated with newly diagnosed active TB cases in both periods

**Period**							
**2001-2004**				**2005-2008**			
**Variables (n)**	**Contacts with newly diagnosed active TB case** **(n = 16)**	***p value***	**OR (CI 95%)**	**Variables (n)**	**Contacts with newly diagnosed active TB case (n = 35)**	***p value***	**OR (CI 95%)**
Index case AFB positive (n =193)	9 (56.2%)	0.1	0,4 (0.1-1.1)	Index case AFB positive (n = 287)	22 (62.9%)	0.2	0,6 (0.3-1.2)
Household contact (n = 620)	14 (87.5%)	0.5	1,9 (0.4-8.7)	Household contact (n = 761)	24 (68.6%)	0.3	1,4 (0.7-3.0)
Smoking contact (n = 81)	1 (6.2%)	0.9	0,5 (0.07-4.4)	Smoking contact (n = 88)	6 (17.1%)	0.04	2,7 (1.1-6.8)
Contacts living in poor areas (slum) (n = 111)	10 (62.5%)	<0.001	9.4 (3.3-26.5)	Contacts living in poor areas (slum) (n = 205)	11 (36.7%)	0.01	2,6 (1.2-5.7)
Contacts prisoners/previous hospitalization (n = 26)	2 (12.5%)	0.1	4,1 (0.9-19.3)	Contacts prisoners/previous hospitalization (n = 35)	1 (2.9%)	0.6	1.0 (0.1-7.7)

Excluded from the table are injecting drug use, use of non-injectable drugs, diabetes “mellitus”, and use of immunosuppressive drugs because no contact was diagnosed with TB. Statistical test used was Chi-square.

**Table 3:** Variables associated with latent TB infection (LTBI) in both periods

**2001-2004**				**2005-2008**			
**Variables (n)**	**Contacts with LTBI** **(n = 496)**	***p value***	**OR (CI 95%)**	**Variables (n)**	**Contacts with LTBI** **(n = 877)**	***p value***	**OR (CI 95%)**
Index case AFB positive (n = 193)	356 (71.8%)	0.001	0.5 (0.3-0.7)	Index case AFB positive (n = 287)	665 (75.8%)	<0.001	1.7 (1.3–2.3)
Index case HIV positive (n = 4)	12 (2.4%)	0.2	0.4 (0.1-1.3)	Index case HIV positive (n = 5)	16 (1.8%)	0.1	0.4 (0.1–1.1)
Household contact (n = 620)	387 (78%)	0.7	0.9 (0.6-1.3)	Household contact (n = 761)	547 (62.4%)	0.07	1.2 (0.9-1.6)
Smoking contact (n = 81)	54 (10.9%)	0.8	1.0 (0.6-1.8)	Smoking contact (n = 88)	82 (9.4%)	<0.001	9.5 (2.9 – 30.5)
Contacts living in poor areas (slum) (n = 111)	71 (15.5%)	0.6	1.1 (0.7-1.8)	Contacts living in poor areas (slum) (n = 205)	141 (17.7%)	0.9	0.9 (0.6-1.3)
Contacts prisoners/previous hospitalization (n = 26)	22 (4.5%)	0.1	2.6 (0.8-7.6)	Contacts prisoners/previous hospitalization (n =35)	20 (2.3%)	0.3	0.6 (0.2-1.3)
Contacts with alcohol abuse (n = 18)	13 (2.6%)	0.9	1.2 (0.4-3.4)	Contacts with alcohol abuse (n = 45)	44 (5%)	<0.001	14.9 (2.0-109)

Excluded from the table are injecting drug use, use of non-injectable drugs, diabetes “mellitus” and use of immunosuppressive drugs because no contact was diagnosed with LTBI. Statistical test used was Chi-square.

## Discussion

Our findings confirm the importance of screening for active TB and LTBI among adult close contacts of TB cases with no known additional risk factors in a setting with a moderate incidence of TB. The prevalence rate of newly diagnosed TB cases among contacts in 2001–2004 (2%; 1,975/100,000 habitants) and in 2005–2008 (2.7%; 2,442/100,000 habitants) was similar to the prevalence of active TB reported by a meta-analysis (41 articles included in a systematic review) that determine the yield of contact investigation in resource limited settings and found a prevalence of 2.3% (95% CI 2.1–2.5) for bacteriological confirmed active TB cases [14]. Our findings were also similar to those found in a more recent meta-analysis that included 70 studies from low and middle-income settings in which the prevalence of microbiologically proven TB among contacts was 3.1 (95% CI 2.2–4.4) [15]. Although the prevalence rates are similar, it should be noted that the definitions of “contact” varied considerably between the studies analyzed in both meta-analyses. Interestingly, the prevalence of newly diagnosed TB cases (2.7%, 35/1,310) among close contacts in the second period of the present study was the same prevalence of TB cases (2.7%, 15/542) among respiratory symptomatic patients with cough for one week or more who sought care in a Primary Health Center, as reported in a study conducted in the same city of Rio de Janeiro [16]. It should be pointed out that the passive case finding among respiratory symptomatic subjects spontaneously attending Primary Health facilities is the current strategy for TB case finding in Brazil. Furthermore, with exception of few reference Centers for TB like the TB Clinic of IDT/UFRJ, investigation for active TB among close contacts is not routinely conducted, although it is suggested in the National Program of TB [4].

Although the total number of contacts identified during the 2005–2008 (n = 1,310) was higher than in 2001–2004 (n = 810), the NNS for newly diagnosed TB (50 *versus* 37) and the NNCT (16 *versus* 10) decreased from the first to the second period. A review recently published by the World Health Organization (WHO) summarizes the weighted average NNS to find one case of TB in different risk groups in different epidemiological situations [2]. In settings with moderate incidence (30–100 new cases/100,000 inhabit) like Rio de Janeiro city, the average NNS (40; ranging 7–355) was 20% lower than the NNS found in the first period (NNS = 50) and similar to the NNS found in the second period of our study (NNS = 37). The significant reduction in the NNS in the second period may suggest that the less conservative definition of “close contact” applied during this period may be more effective in identifying new cases of TB than the definition of “close contact” used in the first period of this study.

The only variable associated with a new diagnosis of active TB among contacts in both periods was “living in a slum” and variables associated with LTBI were an AFB positive index case, smoking and alcohol abuse (Tables 2 and 3). Interestingly the AFB of the index case and the variable “living in same house” were not associated with active TB diagnosis. On the other hand, in our sample, a positive AFB smear in the index case in both periods, as well as smoking and alcohol abuse in the second period were identified as independent factors associated with the prevalence of LTBI. Smoking and alcohol abuse have been associated with LTBI in specific high risk groups such as adult prisoners, nursing home residents and immigrants from countries with high prevalence of TB disease [17-20]. In fact, a meta-analysis showed that smoking (p < 0,001, OR = 2.1 95% CI 1.4 -3.1) and alcohol abuse (p = 0.001 OR = 4.7 95% CI 1.8-9.8) were independent factors associated with LTBI [21]. A study conducted by Rose and colleagues evaluated the prevalence of LTBI among 1,590 contacts of pulmonary TB patients and found that a higher prevalence of LTBI was associated with AFB positivity and “living in same house” group [7]. However, while the AFB of the index case was associated with LTBI among younger contacts, intimacy of exposure was the variable associated with LTBI among contacts older than 30 years [7]. Furthermore, a study conducted in Pakistan (1961) also found similar prevalence of LTBI (60% *versus* 61%) among contacts of index cases with AFB positive (531 contacts/100 cases) and AFB negative smears (460 contacts/100 cases) [22].

The prevalence of LTBI in both samples of our study (62% and 69%) were greater than the prevalence of LTBI among contacts reported by a study conducted in Botswana and a meta-analysis of studies conducted in low–middle income countries (45.3; 95% CI 40.6–50,1 and 51.4% ; 95% CI 50.6–52,2) [13,14].

The higher level of TB incidence (94/100,000) during the first period of the study (2001–2004) when compared to the second period of the study (2005–2008) could correspond to higher rates of disease transmission which potentially generated a larger population of latently infected individuals that could have eventually resulted in the higher yield of contact-tracing in the second period of the study. However, the incidence rate of TB in years prior (1997–2000) to the study was even higher (110/100,000) and the detection rate of active TB and LTBI during the second period of the study was higher than during the first one [9]. This suggests that the higher level of detection of active and latent TB in the second period of study was the consequence of the less conservative approach used in this period of the study than the impact of higher TB incidence rate in the first period of study.

This study has important limitations. Although the setting is a clinical research site, this is a retrospective study and the evaluation of study subjects and the data collection were conducted under pragmatic clinical ‘non-study’ conditions. The allocation of contacts in both groups (enclosed space with a TB index case for ≥ 100 hours/total period or ≥ 4 hours/week) was not random. However, to our knowledge, this is the first manuscript comparing pragmatically two different definitions of “close contact”. There was an important loss (20-25%) of subjects (index cases and contacts) in both periods. However, the sample studied is still notable and allows important conclusions and analysis. The design of the study does not allow the assessment of the exact duration of exposure (in hours) related to the highest detection of LTBI or newly active TB. However in clinical practice, an individual who has 4 h/week of exposure to a pulmonary TB patient could have a total of 100 h of exposure if the exposure to index case occurred over 7 months. So, this study analyzed the impact of an operational change (definition of exposure to an active pulmonary TB patient) and not the duration of exposure (in hours) itself. Our study population only included close contacts ≥ 15 years old. Based on this, the real prevalence of close contacts can be underestimated. The index cases of our sample were, mostly subjects enrolled in Clinical Research trials of TB in our TB Clinic and were people from the community and with no initially known risk factors for TB. The study findings are restricted to the data domain in terms of population demographics as well as TB incidence rate and further analysis may be required to expand the domain of results to a more generalized setting.

## Conclusions

The findings of this study suggest that the change in the operational definition of a close contact to 4 hours/week increased the detection of news cases of active TB and LTBI, as well as decreased TB NNS (from 50 to 37) and NNCT (from 16 to 10) for active TB case. New studies including cost effectiveness are needed in order to confirm the effectiveness of this strategy

## Competing interests

The authors declare that they have no competing interests.

## Authors’ contributions

CL participated in the study design of the study, carried out the data collection, performed the statistical analysis and drafted the manuscript. MCC participated of the statistical analysis and helped to draft the manuscript. AE participated in the study design of the study and helped to draft the manuscript. FCQM participated of the statistical analysis and helped to draft the manuscript. MBC conceived and coordinated the study, participated in the study design and of its statistical analysis and helped to draft the manuscript. All authors read and approved the final version of the manuscript.

## Pre-publication history

The pre-publication history for this paper can be accessed here:

http://www.biomedcentral.com/1471-2466/14/133/prepub

## References

[B1] World Health Organization Guidelines for intensified case-finding and isoniazid preventive therapy for people living with HIV in resource constrained settings 2011 Geneva: Department of HIV/AIDS, Stop TB Department http://whqlibdoc.who.int/publications/2011/9789241500708_eng.pdf

[B2] World Health Organization Systematic screening for active tuberculosis: principles and Recommendations 2013 Geneva: World Health Organization (WHO) (WHO/HTM/TB/2013.04). http://apps.who.int/iris/bitstream/10665/84971/1/9789241548601_eng.pdf 25996015

[B3] Conde MB Melo FAF Marques AM Cardoso NC Pinheiro VG Dalcin PT Machado Junior A Lemos AC Netto AR Durovni B Sant’Anna CC Lima D Capone D Barreira D Matos ED Mello FC David FC Marsico G Afiune JB Silva JR Jamal LF Telles MA Hirata MH Dalcolmo MP Rabahi MF Cailleaux-Cesar M Palaci M Morrone N Guerra RL Dietze R III Brazilian Thoracic Association Guidelines on tuberculosis J Bras Pneumol 2009 35 1018 1048 19918635 10.1590/s1806-37132009001000011

[B4] Ministério da Saúde do Brasil Manual de Recomendações para o Controle da Tuberculose no Brasil 2011 Brasília: Ministério da Saúde do Brasil http://portal.saude.gov.br/portal/arquivos/pdf/manual_de_recomendacoes_tb.pdf

[B5] Jasmer RM Nahid P Hopewell PC Clinical practice. Latent tuberculosis infection N Eng J Med 2002 347 1860 1866 10.1056/NEJMcp021045 12466511

[B6] Small PM Fujiwara PI Management of tuberculosis in the United States N Eng J Med 2001 345 189 200 10.1056/NEJM200107193450307 11463015

[B7] Rose CE Zerbe GO Lantez SO Bailey WC Establishing Priority during investigation of tuberculosis contacts Int J Tuberc Lung Dis 1979 119 603 609 10.1164/arrd.1979.119.4.603 443630

[B8] Sterling TR Villarino ME Borisov AS Shang N Gordin F Bliven-Sizemore E Hackman J Hamilton CD Menzies D Kerrigan A Weis SE Weiner M Wing D Conde MB Bozeman L Horsburgh R Jr Chaisson RE Three Months of Rifapentine and Isoniazid for Latent Tuberculosis Infection N Engl J Med 2011 365 23 2155 2166 22150035 10.1056/NEJMoa1104875

[B9] Portal da Saúde [homepage on the Internet] TUBERCULOSE - Casos confirmados notificados no Sistema de Informação de Agravos de Notificação - SINAN Net 2013 Brasília: Ministério da Saúde do Brasil http://dtr2004.saude.gov.br/sinanweb/tabnet/dh?sinannet/tuberculose/bases/tubercbrnet.def

[B10] American Thoracic Society Target tuberculin testing and treatment of latent tuberculosis infection Am J Respir Crit Care Med 2000 161 221 247 10.1164/ajrccm.161.supplement_3.ats600 10764341

[B11] Center for Diseases Control Guidelines for the investigation of contacts of persons with infections tuberculosis 2002 Atlanta: Center for Diseases Control and Prevention (CDC) http://www.cdc.gov/mmwr/pdf/rr/rr5415.pdf

[B12] Kranzer K Houben RM Glynn JR Bekker LG Wood R Lawn SD Yield of HIV-associated tuberculosis during intensified case finding in resource-limited settings: a systematic review and meta-analysis Lancet Infect Dis 2010 10 93 102 20113978 10.1016/S1473-3099(09)70326-3 PMC3136203

[B13] Puryear S Seropola G Ho-Foster A Arscott-Mills T Mazhani L Firth J Goldfarb DM Ncube R Bisson GP Steenhoff AP Yield of contact tracing from pediatric tuberculosis index cases in Gaborone Botswana Int J Tuber Lung Dis 2013 17 8 1049 1055 10.5588/ijtld.12.0933 PMC6219389 23827029

[B14] Morrison J Pai M Hopewell PC Tuberculosis and latent tuberculosis infection in close contacts of people with pulmonary tuberculosis in low-income and middle-income countries: a systematic review and meta-analysis Lancet Infect Dis 2008 8 6 359 368 18450516 10.1016/S1473-3099(08)70071-9

[B15] Fox GJ Barry SE Britton WJ Marks GB Contact investigation for tuberculosis: a systematic review and meta-analysis Eur Respir J 2013 41 140 156 22936710 10.1183/09031936.00070812 PMC3533588

[B16] Bastos LG Fonseca LS Mello FC Ruffino-Netto A Golub JE Conde MB Prevalence of pulmonary tuberculosis among respiratory symptomatic subjects in an out-patient primary health unit Int J Tuberc Lung Dis 2007 11 2 156 160 17263285

[B17] Anderson RH Sy FS Thompson S Addy A Cigarette smoking and tuberculin skin test conversion among incarcerated adults Am J Prev Med 1997 13 3 175 181 9181204

[B18] Nisar M Williams CS Ashby D Davies PD Tuberculin testing in residential homes for the elderly Thorax 1993 48 1257 1260 8303634 10.1136/thx.48.12.1257 PMC464987

[B19] Plant AJ Wartkins RE Gushualak B Rouke TO Jones W Streeton J Sang D Predictores of tuberculin reactivity among prospective Vietnamese migrants: The effect of smokimg Epidemiol Infect 2002 128 37 45 11895089 10.1017/s0950268801006434 PMC2869793

[B20] Solsona J Caylà JA Nadal J Bedia M Mata C Brau J Maldonado J Milà C Alcaide J Altet N Galdós-Tangüis H Screening for tuberculosis upon admission to shelters and free-meal services Eur J Epidemiol 2001 17 123 128 11599684 10.1023/a:1017580329538

[B21] Slama K Chiang CY Enarson DA Hassmiller K Fanning A Gupta P Ray C Tobacco and tuberculosis: A quantitative Systematic review and meta-analysis Int J Tuberc Lung Dis 2007 11 1049 1061 17945060

[B22] Yad MI Observations on the examination of contacts of tuberculosis patients - a 15 year study (1953–67) Bull Int Union Tuberc 1974 49 suppl 1 261 266 4468015

